# South West Orthopaedic Club

**Published:** 1974-10

**Authors:** 


### South West Orthopaedic Club

A Meeting of the Club was held at Yeovil District Hospital on Saturday, 11th May 1974.

There was a very excellent demonstration of Clinical Cases. These included several patients with severe flaying injuries of the forearm and hand which occurred to workers in the leather industry. Most of these had been treated very successfully with abdominal skin flaps, together with later tendon reconstructive surgery. Other injuries of the forearm and hand were shown, together with one patient who had a pollicisation for an absent thumb. Other patients demonstrated injuries to the knee and arthroplasty of the knee joint and further patients demonstrated unusual pathology of the hip joint. There was also an X-ray quiz, and an optional tour of the wards.

At the end of the morning **Mr. P. J. M. Morrison** read a paper on brucellosis of the hip joint. He noted that brucellosis in cattle was very common, particularly in North Somerset, and indeed was not uncommon in farmers dealing with these cattle. Patients below the age of sixteen were rarely affected and, although arthralgia was common, radiological bone involvement other than in the spine was rare. He illustrated his talk with two children with brucellosis of the hip, both showing radiological changes in the metaphysis at the upper end of the femur. The diagnosis is confirmed by a positive culture, but he also stressed the value of serological tests. The cases were treated, one with tetracycline and the other with septrim, both successfully.

Three papers were read during the afternoon session.

**Mr. W. Bunting** of Taunton read a paper on Pes Anserinus Transfer. He described the mechanism of meniscal damage during rotation injuries of the knee and associated injuries of the medial collateral ligaments. If the rotational stress is excessive then the medial collateral ligament is ruptured. Patients with tears of the medial collateral ligament and rotary instability particularly complain of the inability to "cut" when running. The physical signs included a positive anterior draw sign at the knee when the joint was in external rotation, this being abolished by internal rotation of the joint. He described, with the aid of excellent slides, the operation of Pes Anserinus Transfer and the good results in his sixteen patients.

**Dr. John Feagon,** of San Francisco, started his paper with an excellent film showing the mechanism of the football injury which is common in America. He followed this with a collection of slides, many of which were very amusing, showing the mechanism of injury and his method of treating the injury, based upon the work of Slocum. He particularly stressed the importance of repairing the posterior oblique capsular ligament through a sail incision soon after the injury. If it were necessary to perform this repair at a later stage it was imperative to always support it with a muscle transfer, namely a Pes Anserinus Transfer. After operation the knee is splinted in a sugar tongs splint for two weeks, followed by a hinge case for a further four to six weeks. The contra-indications to this operation were a loose jointed patient, a valgus knee or osteoarthrosis.

Following these papers a lively discussion ensued, during which many points were raised including the value of an arthrogram under stress and the advisability of early operation following this injury.

After tea **Mr. Robert Owen** of Liverpool presented his current views on the management of the unstable spine in childhood. He spoke in general about the many and difficult spinal deformities, particularly occurring in the congenital group. He felt that operations on the unstable spine were more akin to salvage operations than true corrective procedures. He stressed also the association of other congenital defects, for example diaphragmatic hernias. He drew particular attention to the presence of a bony bar on the concave side of the congenital scoliosis. He stressed the value of tomography to demonstrate this. Early excision of the bony bar is usually indicated, but care should be taken when doing this as damage to the main artery can lead to ischaemia. One of the problems with children who have had treatment of a meningo-myelocele is a severe increasing lordosis which is extremely difficult to treat. He also drew attention to the splint syndrome, this being kinking of the duodenum where it is put upon by the supra-mesenteric pedicle which occurs when the patient is stretched as after the insertion of Harrington's rods. In Liverpool they are forming a synostosis of the ribs on the convex side of the curve in an attempt to provide a correction. So far the results of this have been encouraging. Mr. Owen mentioned briefly infantile idiopathic scoliosis and its association with plagiocephaly. 92% of these children resolve spontaneously, but the remaining 8% often develop very vicious curves. He discussed recent views on the occurrence of this, which is more common in British children than American, thought to be due to the British habit of laying their children on their side whereas the Americans place their children prone on hard mattresses. Mr. Owen also illustrated the use of Dwyer rods, noting that preliminary skeletal traction usually with crutchfield tongs through the skull was advantageous. Kyphosis he had treated in a few patients with a pedicle rib graft which was strutted across the front of the thoracal lumbar vertebral column. Again so far the results have been encouraging. Mr. Owen stressed that his talk had been brief, but that he had attempted to present his views on some of the more difficult problems.

A discussion after the lecture continued for some considerable time and it was clear that the audience had been greatly stimulated by this lecture.

After the Meeting a dinner was held which was attended by members and their guests.

The next meeting of the Club will be a joint meeting with the Royal College of Surgeons of England in Exeter on December 13/14th 1974.

